# Renin angiotensin system-induced muscle wasting: putative mechanisms and implications for clinicians

**DOI:** 10.1007/s11010-024-05043-8

**Published:** 2024-05-29

**Authors:** Baris Afsar, Rengin Elsurer Afsar, Yasar Caliskan, Krista L. Lentine, John C. Edwards

**Affiliations:** 1 Department of Nephrology, School of Medicine, Suleyman Demirel University, Isparta, Turkey; 2 Division of Nephrology, School of Medicine, Saint Louis University, St. Louis, MO USA

**Keywords:** Angiotensin II, Muscle, Muscle atrophy, Renin angiotensin system, Sarcopenia

## Abstract

Renin angiotensin system (RAS) alters various mechanisms related to muscle wasting. The RAS system consists of classical and non-classical pathways, which mostly function differently. Classical RAS pathway, operates through angiotensin II (AngII) and angiotensin type 1 receptors, is associated with muscle wasting and sarcopenia. On the other hand, the non-classical RAS pathway, which operates through angiotensin 1–7 and Mas receptor, is protective against sarcopenia. The classical RAS pathway might induce muscle wasting by variety of mechanisms. AngII reduces body weight, via reduction in food intake, possibly by decreasing hypothalamic expression of orexin and neuropeptide Y, insulin like growth factor-1 (IGF-1) and mammalian target of rapamycin (mTOR), signaling, AngII increases skeletal muscle proteolysis by forkhead box transcription factors (FOXO), caspase activation and muscle RING-finger protein-1 transcription. Furthermore, AngII infusion in skeletal muscle reduces phospho-Bad (Ser136) expression and induces apoptosis through increased cytochrome c release and DNA fragmentation. Additionally, Renin angiotensin system activation through AT1R and AngII stimulates tumor necrosis factor-α, and interleukin-6 which induces muscle wasting, Last but not least classical RAS pathway, induce oxidative stress, disturb mitochondrial energy metabolism, and muscle satellite cells which all lead to muscle wasting and decrease muscle regeneration. On the contrary, the non-classical RAS pathway functions oppositely to mitigate these mechanisms and protects against muscle wasting. In this review, we summarize the mechanisms of RAS-induced muscle wasting and putative implications for clinical practice. We also emphasize the areas of uncertainties and suggest potential research areas.

**Graphical abstract:**

Classical and non-classical renin angiotensin systems (RAS) play opposing roles in muscle wasting. Classical RAS system operates through Angiotensin (Ang)I/ACE/AngII)/Angiotensin Type 1 Receptor (AT1R) and induces muscle wasting by mechanisms including inducing anorexia, ubiquitin–proteasome system (UPS), apoptosis, inflammation, oxidative stress, mitochondrial dysfunction, albuminuria, fibrosis (increasing transforming growth factor beta, connective tissue growth factor) and decrease insulin-like growth factor 1 (IGF-1) signaling, vitamin D and satellite cell function. Non-classical RAS system operates through Angiotensin1/ACE2/Ang (1–7)/Mas Receptor and have opposite actions to classical RAS system and protects against muscle wasting.

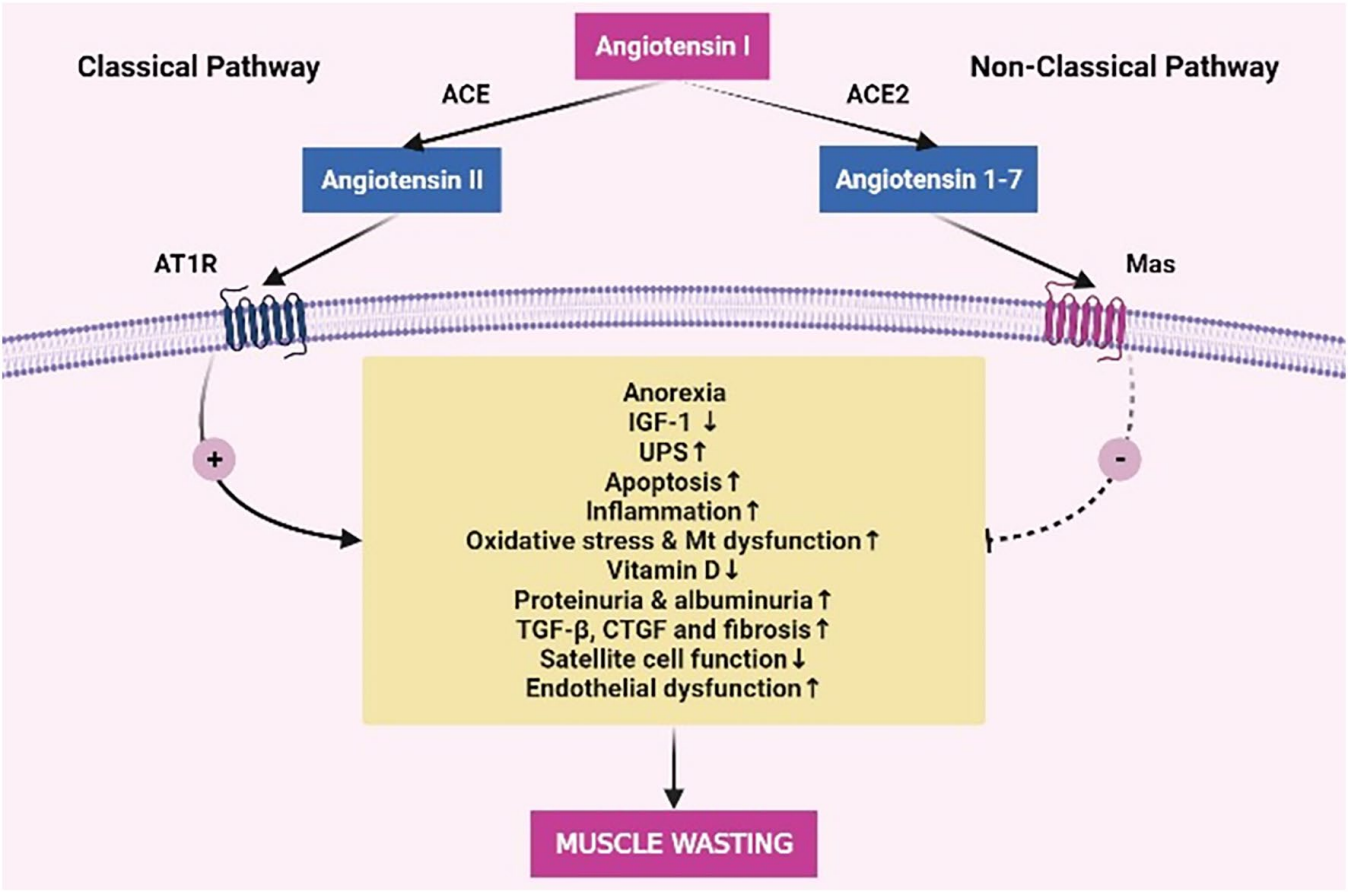

## Introduction

Renin angiotensin system (RAS) plays a fundamental role in the regulation of blood pressure, and fluid and salt balance. Apart from these roles, there is growing evidence showing that RAS is involved in various mechanisms of muscle wasting. The RAS system consists of classical and non-classical pathways, which mostly function differently. The classical RAS pathway operates via angiotensin II (AngII) and angiotensin Type 1 receptor (AT1R), and is associated with muscle wasting. On the other hand, the non-classical RAS pathway operates via Ang (1–7), angiotensin Type 2 receptor (AT2R) and Mas receptors, and is protective against muscle wasting. AngII might induce muscle wasting by various mechanisms, which include insulin signaling, protein degradation, appetite regulation, apoptosis, inflammation, oxidative stress, and transforming growth factor beta (TGF-β) [1, 2]. AngII reduces food intake and body weight, by decreasing hypothalamic expression of orexin and neuropeptide Y [3], decreases insulin like growth factor (IGF-1) [4], and mammalian target of rapamycin (mTOR) signaling [5] increases skeletal muscle proteolysis by caspase activation [5] and Muscle RING-finger protein-1 (Murf-1) transcription [6]. Furthermore, AngII infusion in skeletal muscle reduces phospho-Bad (Ser136) expression [5], induces apoptosis [7] and DNA fragmentation [5]. Additionally, Renin angiotensin system activation through AT1R AngII stimulates interleukin-6 [8] and tumor necrosis factor-α [9], and which induces muscle wasting [10], Last but not least classical RAS pathway, induce muscle oxidative stress [11], disturb mitochondrial energy metabolism in muscle [12], and muscle satellite cells [13] which all lead to muscle wasting and decrease muscle regeneration. These mechanisms act synergistically with each other in vivo. On the contrary, the non-classical RAS pathway, which operates through angiotensin 1–7 and Mas receptor, try to neutralize these detrimental pathologic process thus protective against sarcopenia as explained in detail below. In the current review, we summarize the mechanisms of RAS-induced sarcopenia both in vivo and in vitro. Our first aim is to draw attention to the RAS system as an important role player in sarcopenia. Secondly, we aim to identify research deficits and suggest potential research areas in the context of sarcopenia.

## Renin angiotensin system-induced muscle wasting: suggested mechanisms

### Renin angiotensin system, anorexia and body weight

Appetite is one of the most important factors influenced by RAS. Experimentally, in rats, a 1-week infusion of AngII reduces body weight by 18–26% compared to sham, via reduction in food intake. Losartan (an AT1R antagonist), but not hydralazine (a vasodilator), restores losses in body weight with comparable effects on blood pressure, suggesting that AngII has appetite regulating effects independent of blood pressure [14]. Furthermore, peripheral AngII infusion in mice (1 g/kg per min) decreases hypothalamic expression of orexin and neuropeptide Y (NPY), and food intake at 6 h compared to placebo, despite not changing peripheral leptin, adiponectin, glucagon-like peptide, ghrelin, or cholecystokinin levels. Importantly, candesartan or deletion of AT1R decrease NPY and orexin reduction in hypothalamic cultures in vivo. In addition, cerebral ventricular AngII infusion (50 ng/kg per min) reduces food intake, which is completely prevented by candesartan [3]. In Sprague–Dawley rats, AngII infusion impairs appetite and decreases body weight by inducing wasting predominantly in adipose tissue [15]. These findings are in accordance with the evidence showing that AT1R knockout mice are hyperphagic [16], and suggest that RAS system is directly involved in appetite regulation.

### Renin angiotensin system, insulin, IGF-1 and insulin resistance

**Fig. 1:**
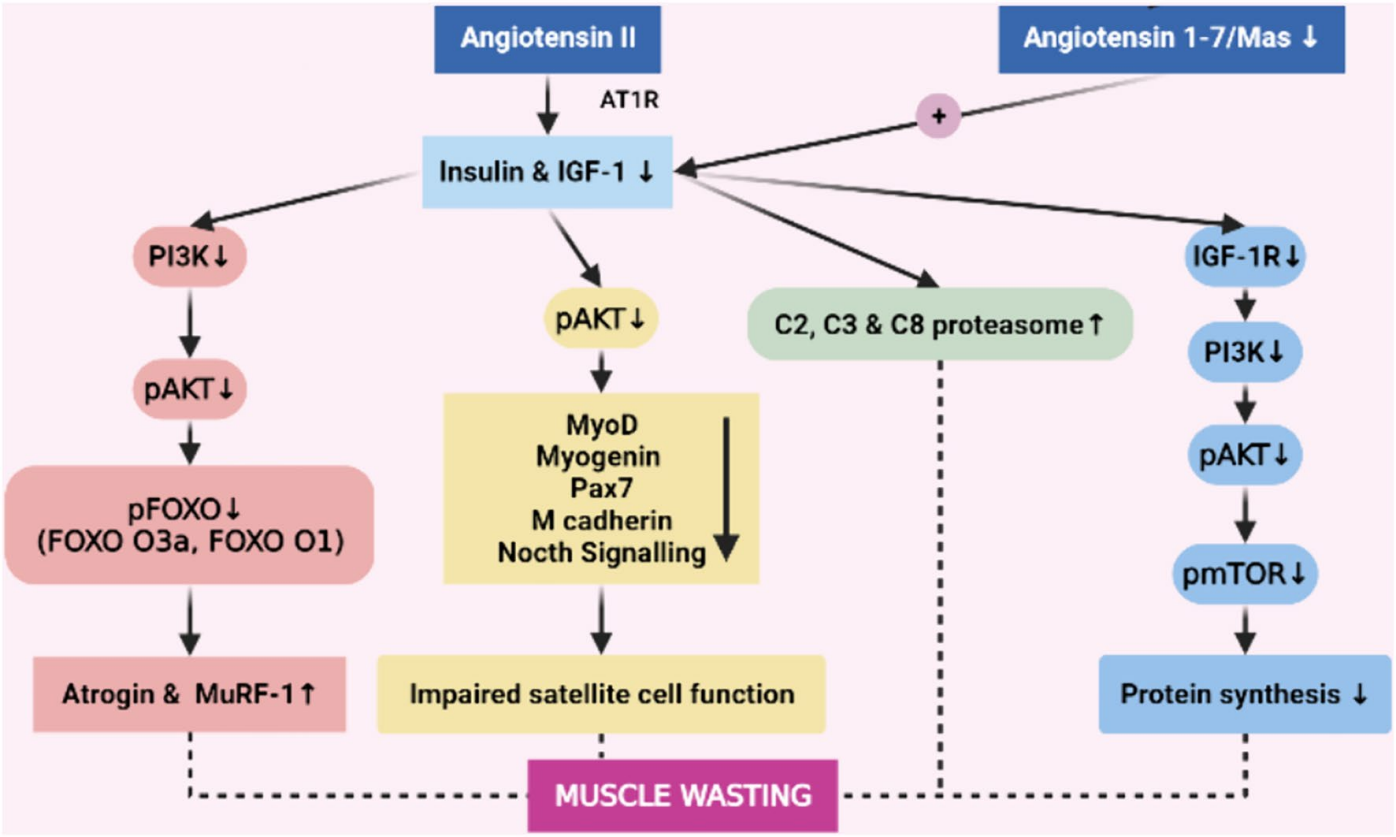
Insulin like growth factor 1 (IGF-1) is inhibited by angiotensin II (AngII). When Insulin-IGF-1 signaling decreased; phosphorylated protein kinase B (pAKT) decreases, which have various consequences. Normally, pAKT phosphorylates and inactivates forkhead box transcription factors (FOXOs). When pAKT is decreased, FOXO inactivation decreases, resulting in increased atrogin and muscle RING-finger protein-1 (Murf-1), and subsequent muscle wasting. Decreased pAKT impairs satellite cell function and muscle regeneration capacity by decreasing myoD, myogenin and M cadherin proteins, Pax 7 (a transcription factor for myogenesis) and Notch signaling. Decreased pAKT decreases phosphorylated mammalian target of rapamycin (pmTOR) and protein synthesis in muscle. Decreased insulin and IGF-1 activates proteasomes and results in muscle protein breakdown. Ang1-7/Mas increases Insulin/IGF-1 and offsets muscle wasting

In the skeletal muscle, the binding of Insulin-like growth factor 1 (IGF-1) or insulin to its receptor activates two major signaling pathways: the Ras-Raf-MEK-ERK and the PI3K/Akt/mTOR pathways [11]. AngII, by decreasing insulin and IGF-1, affects various downstream cellular signaling pathways, which have impacts on muscle wasting (Fig. 1). Studies suggest that decreased activity of the IGF-1/PI3K/Akt signaling can lead to muscle atrophy [17, 18], and AngII has shown to decrease IGF-1/PI3K/Akt signaling [4]. In Sprague–Dawley rats, AngII infusion (500 ng/kg/min) for up to 14 days reduces plasma IGF-1 levels (56% and 41% decrease at 1 and 2 weeks, respectively) [14], and similar findings are observed in C57BL/6 mice [5]. Blink et al. showed that AngII infusion decreases IGF-1, IGF binding protein-3 and IGF binding protein-5 mRNA expression in the gastrocnemius muscle and increases skeletal muscle protein breakdown. Surprisingly, co-infusing systemic IGF-1 and AngII, does not prevent muscle loss despite increased circulating IGF-1 levels, suggesting that local muscle IGF-1, but not systemic IGF-1, may be of importance to prevent muscle wasting [7]. Indeed, this hypothesis is confirmed by showing that the specific targeted expression of IGF-1 in skeletal muscle completely inhibits AngII-induced weight loss and muscle wasting [5].

Mammalian target of rapamycin (mTOR) signaling is also important for IGF-1 actions in muscle. IGF-1, by binding to IGF-1 receptor, increases phospho-mTOR induced muscle hypertrophy, but this effect is ameliorated by AngII infusion [5]. In mice, immobilization results in loss of tibialis anterior muscle mass through down-regulation of the IGF-1/Akt/mTOR pathway. In these mice, losartan increases the expressions of phospho-Akt, phospho-Forkhead box protein class O (FOXO) 3a (FOXO3a) and phospho-mTOR, and ameliorates muscle wasting [19]. IGF-1 may also prevent against AngII-induced wasting by an Akt and a FOXO-1-dependent signaling pathway that results in inhibition of atrogin-1 [20]. Furthermore, AngII-induced protein degradation in murine myotubes is completely attenuated by IGF-1 [21]. IGF-1 also suppresses C-2, -3 and -8 proteasome subunits and ubiquitin mRNAs in the skeletal muscle of rats treated with dexamethasone [22]. Thus, all of the aforementioned evidence suggests that AngII induces muscle protein breakdown by diminishing IGF-1 signaling.

Additionally, RAS system also involves AT2R and Mas receptors, which generally have opposing functions to classical RAS. In the disuse muscle atrophy model, the functions of Ang-(1–7) and the Mas receptor in wild-type and Mas-knockout (Mas-KO) mice were studied for 1 and 14 days. Importantly, Ang (1–7) improves muscle strength, and reduces myosin heavy chain, atrogin-1 and muscle ring finger-1 (MuRF-1) levels. Moreover, Ang (1–7) increases IGF-1/IGFR-1/Akt pathway signaling through IGFR-1 and increases Akt phosphorylation with concomitant activation of two downstream targets of Akt, namely p70S6K and FOXO3, via the Mas receptor. These anti-atrophic effects of Ang (1–7) are not observed in Mas-KO mice, indicating a critical participation of the Mas receptor for decreasing muscle wasting [23].

### Ubiquitin-proteasome pathway

The ubiquitin-proteasome system (UPS) is one of the major contributors to muscle protein degradation. In rats, AngII infusion increases muscle protein breakdown, which is not abolished by inhibition of lysosomal and calcium-dependent proteases, suggesting a role of non-lysosomal and calcium-independent proteolytic system. However, when proteasome inhibitor is added, protein breakdown decreases sharply, suggesting the role of UPS in the skeletal muscle protein breakdown [7]. Song et al. elegantly demonstrated that AngII infusion in C57BL/6 mice increases skeletal muscle proteolysis by decreasing phosphorylation of Akt, FOXO1, FOXO3, FOXO4, and glycogen synthase kinase-3 beta (GSK3β), and by increasing caspase-3 activation (sixfold), and mRNA levels of atrogin-1 (24-fold) and MuRF-1(fivefold), which are two muscle-specific ubiquitin ligases in muscle atrophy models [5]. Further, studies shed a light on the molecular mechanisms of MuRF-1 regulation in sarcopenia. Bois et al. showed that transcription factor EB (TFEB) -regulator of the human MuRF1 promoter-plays an important role in AngII-induced skeletal muscle atrophy. TFEB binds to specific E-box motifs in the promoter of the lysosomal genes. AngII increases MuRF1 transcription via E-box elements and induces muscle wasting, however, when TFEB is inhibited by siRNA, AngII-induced muscle wasting is prevented. Furthermore, protein kinase D1 (PKD1) is needed for the occurrence of muscle wasting action of TFEB, and mice lacking PKD1 in skeletal myocytes are resistant to AngII-induced muscle wasting [6]. AngI and AngII also stimulate protein expression of 20S proteasome subunits (suggesting high number of proteases), which is attenuated by co-incubation with the angiotensin-converting enzyme (ACE) inhibitor imidaprilat [21]. On the contrary, Cisternas et al. showed that skeletal muscle atrophy induced by AngII is reversed by Ang (1–7) via Mas receptor through decreased atrogin-1 and MuRF-1 expression and increased Akt phosphorylation [24].

### Apoptosis

Angiotensin II increases muscle apoptosis of skeletal muscle by affecting Bad and cytochrome c [5]. Akt promotes cell survival by phosphorylating Bad at Ser136 and inactivates them, which are upstream components of the apoptosis cascade [25, 26]. AngII infusion in skeletal muscle reduces phospho-Bad (Ser136) expression and induces apoptosis through increased cytochrome c release and DNA fragmentation. Moreover, it is shown that spironolactone, an aldosterone antagonist, significantly reduces the skeletal myocyte apoptosis by 79% [27]. Meneses et al. showed that through Mas, Ang (1–7) decreases apoptotic nuclei, expression of caspase-8, caspase-9 activity and Bax/Bcl-2 ratio, which are associated with the attenuation of decrease in fiber diameter and muscle strength [2].

### Inflammation

**Fig. 2:**
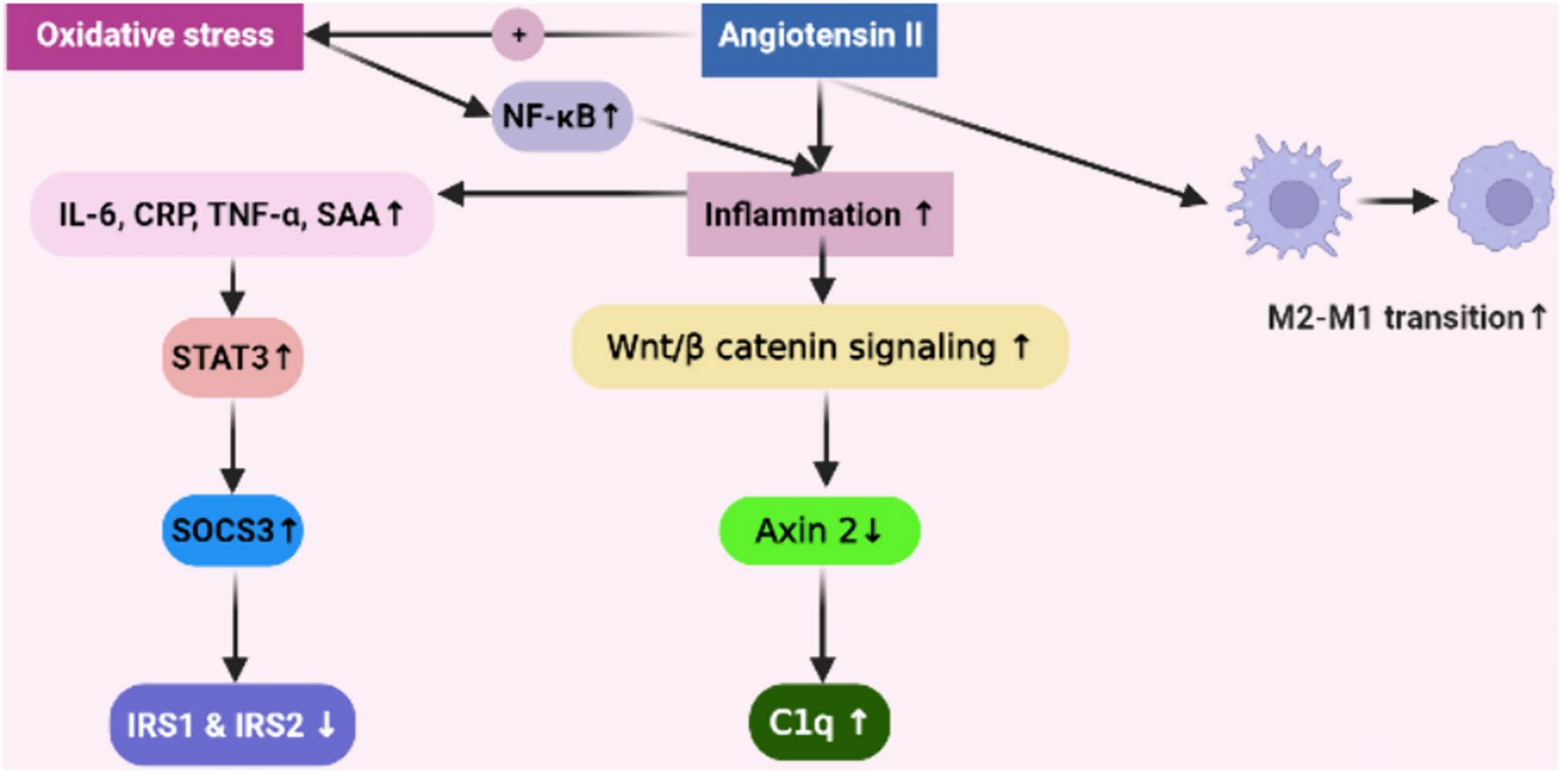
Increased AngII have pro-inflammatory effects. Angiotensin II (AngII) increases oxidative stress, which augments inflammation via nuclear factor kappa B (NF-κB) signaling. AngII directly stimulates tumor necrosis factor alpha (TNF-α), interleukin-6 (IL-6) serum amyloid A (SAA) production, which increase signal transducer and activator of transcription 3 (STAT3) factor. Increased STAT3 boosts muscle suppressor of cytokine signaling (SOCS3) expression, leading to reduced insulin receptor-substrate-1 (IRS-1) and IRS-2 levels in muscle, AngII also activates C1q mRNA expression via increased Wnt/β-catenin signaling causing decreased Axin*2* expression -a down downstream molecule of the Wnt/*β*-catenin signaling pathway- AngII induces M2 macrophage to M1 macrophage polarization, which further increases inflammation

Renin angiotensin system activation through AT1R promotes inflammation. AngII directly stimulates tumor necrosis factor-α (TNF-α) and interleukin-6 (IL-6) production in human peripheral monocytes and ACE inhibitors inhibit lipopolysaccharide-induced TNF-α production [28]. Tumor necrosis factor-α enhances muscle wasting via Nuclear Factor kappa B (NF-κβ) signaling [29], and TNF-α treatment attenuates insulin-stimulated protein synthesis [30]. In mice, AngII infusion elevates levels of IL-6 and serum amyloid A to increase muscle suppressor of cytokine signaling (SOCS) 3 expression, leading to reduced insulin receptor-substrate-1 (IRS-1) levels, and thus induces muscle wasting [31]. These findings confirm previous data suggesting that cytokines, including IL-6, stimulate the expression of SOCSs, via activation of signal transducer and activator of transcription 3 (STAT3) phosphorylation, which in turn stimulates UPS-mediated degradation of IRS-1 and IRS-2 [32, 33]. Yabumoto et al. studied the effects of complement q1 after cryoinjury in tibialis anterior muscle in mice either fed with standard chow or a chow containing irbesartan (20 mg/kg/day), which was started one day prior to cryoinjury operation. In irbesartan-treated mice, there was an anti-inflammatory M2 macrophage shift. Furthermore, irbesartan decreased C1q mRNA expression associated with decreased Wnt/β-catenin signaling and decreased Axin2 expression in macrophages which is a downstream molecule of the Wnt/*β*-catenin signaling pathway (Fig. 2). These beneficial effects of irbesartan is reversed by topical administration of C1q [34].

Inflammation also causes insulin resistance, and it is shown that TNF-α stimulates phosphorylation of IRS-1ser307, leading to suppression of insulin/IGF-1 signaling [35, 36]. This is relevant given that impaired insulin/ IGF-1 signaling is closely linked to activation of muscle protein degradation, as suggested above. Morales et al. showed that endotoxin decreases myofiber and myotube diameters, increases atrogin-1 and MuRF-1 expression and p38 MAPK activation, which are all ameliorated by Ang-(1–7)/Mas axis [37].

### Oxidative stress, energy hemostasis and mitochondrial dysfunction

**Fig. 3:**
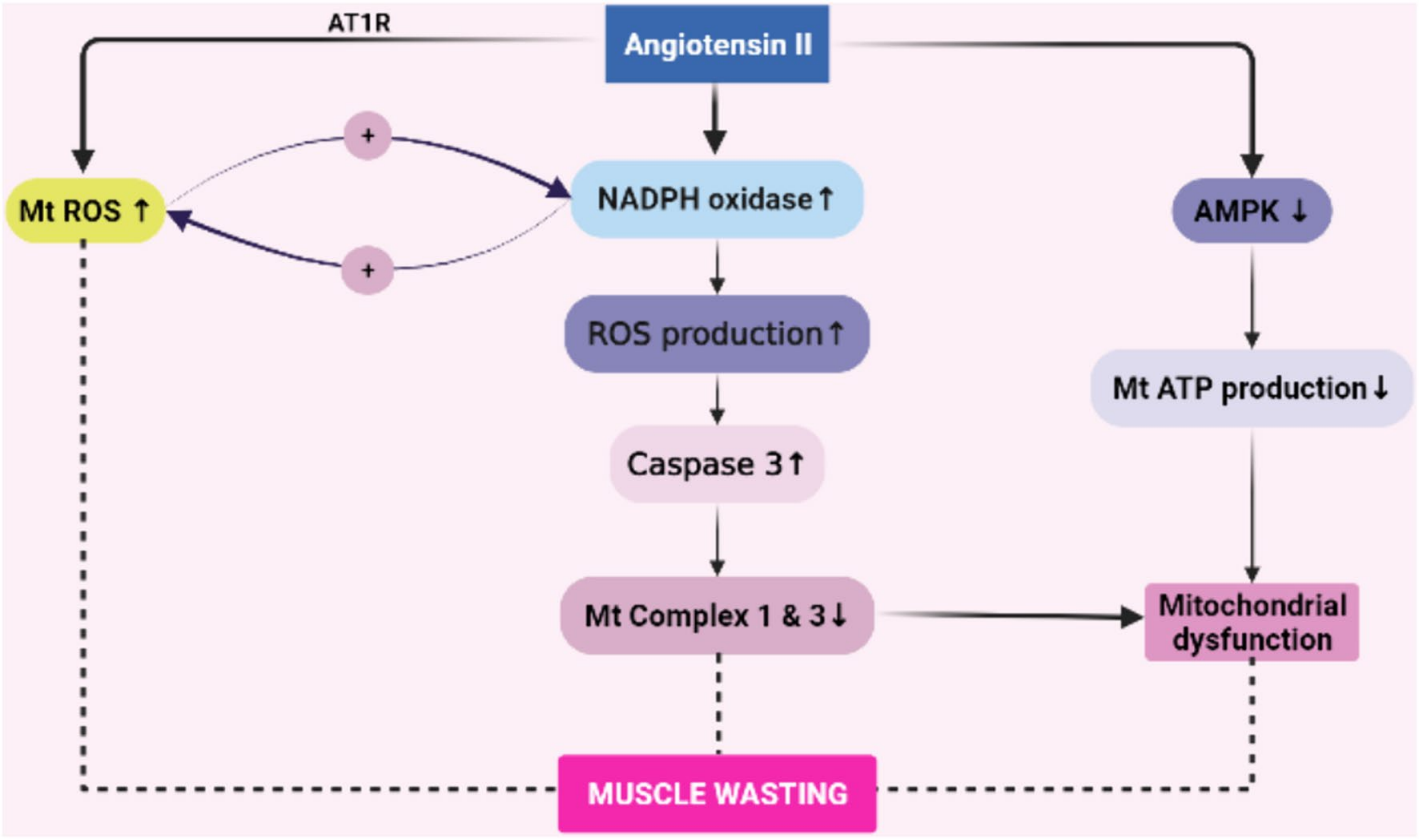
Mitochondrial and nicotinamide adenine dinucleotide phosphate (NADPH)-mediated oxidative stress is increased by angiotensin II (AngII). NADPH dependent reactive oxidative species (ROS) formation and mitochondrial ROS formation are interlinked and stimulate each other in a positive feedback loop. ROS production augments caspase-3 activity, resulting in a decreased mitochondrial complex 1 and 3, mitochondrial dysfunction and muscle wasting. AngII also decreases AMP-activated protein kinase (AMPK), disturbing ATP production in mitochondria and mitochondrial function

Reactive oxygen species (ROS) and mitochondrial dysfunction play an important role in muscle atrophy [11, 38]. In rats, enalapril and losartan both protect against age-related mitochondrial dysfunction and the ultrastructural alterations associated with aging [38]. Oxidative stress activates NF-kB via augmentation of gene expressions of pro-inflammatory cytokines and chemokines. Additionally, inflammation and oxidative stress augment each other [39–41], thus causing a positive feedback loop between inflammation, oxidative stress, RAS activation, which further augments muscle atrophy. Semprun et al. showed that AngII infusion to C57BL/6 J mice induces body and skeletal muscle weight loss along with increased nicotinamide adenine dinucleotide phosphate (NADPH) oxidase-derived superoxide production by 2.4-fold. However, treatment with apocynin, -an NADPH oxidase inhibitor- prevents Ang II-induced superoxide production in skeletal muscle [42]. Similarly, in mice, subcutaneous AngII infusion (1000 ng/kg*/*min) increases NAD(P)H oxidase-derived superoxide caspase-3 and decreases citrate synthase and complex I and III activity in mitochondria [43]. Thus, apart from NADPH induced ROS production, mitochondrial ROS production is also important in the pathogenesis of muscle atrophy. Indeed, NADPH- dependent ROS formation and mitochondrial ROS formation are interlinked and stimulate each other through positive feedback, triggering the cycle of subsequent NADPH oxidase activation [44]. This is important with respect to RAS signaling in the context of muscle wasting as AngII stimulates both NADPH oxidase and mitochondrial ROS production (Fig. 3). Indeed, it is shown that AngII increases ROS generation by enhancing NADPH oxidase activity in myotubes, which are ameliorated both by losartan and apocynin [45].

Angiotensin II also plays a role in muscle wasting by disturbing energy production. During muscle atrophy, energy production in the form of ATP decreases [46]. AngII, by increasing phosphatase 2A catalytic subunit, inhibits AMPK activation in skeletal muscle, which is the main energy sensor of the cell, this inhibition by AngII is reversed by 5-aminoimidazole-4-carboxamide-1-β-D-ribofuranoside -an AMPK activator- which increases cellular ATP levels. Thus, AngII depletes skeletal muscle ATP content likely via induction of mitochondrial dysfunction [47]. These evidences suggest that AngII induces mitochondrial oxidative stress and disturbs mitochondrial energetics, which in turn result in skeletal muscle atrophy (Fig. 3).

### Transforming growth factor beta, connective tissue growth factor and fibrosis

Transforming growth factor beta signaling is augmented during muscle wasting with various consequences. First, increased TGF-β activity inhibits satellite cell activation and myocyte differentiation [48, 49]. Second, TGF-β exerts its effects on muscle by canonical Smad-dependent pathway and by non-canonical Smad-independent pathway. The Smad-dependent pathway causes phosphorylation of Smad2, Smad3, or both, which then binds to Smad4 affecting transcription [50]. The non-canonical pathway operates through MAPK, including extracellular ERK1/2, c-Jun N-terminal kinases, and p38 [51]. Both canonical and non-canonical TGF-β pathways contribute to different mechanisms of sarcopenia by inhibiting myogenic regulatory factors, leading to insufficient muscle regeneration and the increased formation of tissue fibrosis [52–54]. Cohn et al. showed that AT1R blockage by losartan inhibits canonical TGF-β signaling, nuclear Smad2 accumulation and fibrosis in mouse models of Marfan syndrome [55]. In congenital muscular dystrophy, losartan inhibits TGF-β signaling, and downstream phosphorylated Smad2/3 proteins, decreases fibrosis and improves muscle strength. Moreover, losartan activates Smad7 protein, a key negative regulator of TGF-β signaling [56]. Similarly, in laminin-α2-deficient congenital muscular dystrophy, L-158809, a derivative of losartan, decreases TGF-β signaling [57]. Importantly, the atrophic effect of TGF-β1 in myotubes is prevented by Ang (1–7) through Mas receptor. Specifically, Ang (1–7)-Mas receptor axis decreases MuRF-1, protein ubiquination, fiber diameter reduction and ROS production. However, these protective effects are blocked by A779, an antagonist of the Mas receptor [58].

Connective tissue growth factor (CTGF) is another molecule which induces tissue fibrosis by a mechanism involving ERK-1/2 phosphorylation [59]. In myoblasts, fibronectin collagen type III is increased in response to CTGF along with ERK phosphorylation and increased fibrosis. Losartan decreases all these detrimental findings and increases skeletal muscle strength, showing protection against fibrosis [60]. Additionally, in dystrophic mice, Ang (1–7)/Mas signaling inhibits the pro-fibrotic effects mediated by TGF-β. Histological analyses showed that fibronectin and CTGF levels, two markers of fibrosis, decrease but muscular force increases after Ang (1–7) treatment [61].

Myostatin is another member of TGF-β family. Myostatin negatively impacts skeletal muscle mass and growth leading to muscle atrophy through a complex signaling pathway involving Smad, MAPK and Akt [62, 63]. Angiotensin II increases myostatin levels [64]. On the other hand, myostatin-induced decrease in myotube diameter and myofibrillar protein levels and myostatin-induced ROS production, atrogin-1, MuRF-1, and TNF-α gene expressions and NF-κB signaling are restored by Ang (1–7). These protective effects of Ang (1–7) operate through Mas receptor, and they are lost when the Mas receptor antagonist A779 was used [1].

### Satellite cell function

Satellite cells are skeletal muscle-specific stem cells, which have high myogenic potential and self-renewal properties. During a healthy state, the number and regeneration capacity of satellite cells remain almost constant [65]. Following muscle injury, satellite cells are activated and express the MyoD and myogenin transcription factors, leading to cell proliferation and differentiation, respectively. Furthermore, satellite cells express embryonic myosin heavy chain protein leading to myotube formation. Impaired IGF-1 signaling through decreased Akt-dependent phosphorylation is one of the mechanisms for decreased satellite cell function and associated fibrosis [66] (Fig. 1). The canonical RAS system negatively impacts satellite cell function. It is shown that a 7 day AngII infusion decreases expression of satellite proliferation/differentiation markers (MyoD, myogenin, PaxA7, M cadherin, and Notch signaling) in vivo and in vitro and deplete satellite cell pool. AngII also suppresses cyclins involved in cell division process, providing further evidence that Ang II suppresses satellite cell division in regenerating skeletal muscle. These effects of AngII are attenuated either by pharmacologic (with candesartan (10 mg/kg/day)) or genetic (AT1R-null mice) manipulations [13]. Furthermore, Notch signaling activation diminishes the AT1R-mediated anti-proliferative effects of AngII in cultured satellite cells, consistent with the previous findings suggesting that decreased Notch signaling in satellite cells impair proliferative capacity [48]. Kobayashi et al. demonstrated that muscle-derived stem cells treated with losartan is superior to muscle-derived stem cells not treated with losartan in decreasing scar formation, increasing in the number of regenerating myofibers and expression of Smad7 and MyoD [67].

### Vitamin D, renin angiotensin system and muscle wasting

Vitamin D is one of the main hormones regulating muscle physiology [68]. Low levels of vitamin D are related to low muscle mass and function [69]. Indeed, vitamin D has anabolic effects in muscle fibers and deficiency of vitamin D is associated with muscle weakness [70]. It has been known that vitamin D is a negative regulator of RAS and supplementation of vitamin D represses renin expression [71]. Li et al. recently demonstrated one of the most convincing data regarding vitamin D signaling, RAS system and muscle wasting. The study had two arms; a clinical arm analyzed the association between vitamin D level, muscle strength and circulating AngII levels in 1034 participants, and in experimental arm, dexamethasone was applied to induce muscle atrophy in WT and vitamin D receptor (VDR)-null mice, and the mice in which muscle atrophy was induced, were treated with calcitriol for 10 days. In the clinical arm, low muscle strength was associated with low vitamin D and high serum AngII levels. Dexamethasone- and Ang II-induced muscle atrophy was aggravated in VDR null mice, which was mediated by PI3K/Akt/FOXO1 signaling. Importantly, calcitriol treatment increased grip strength, fiber area, and improved fiber type composition by suppressing renin/AngII axis [72].

## Human studies and reflections for clinical management

As aforementioned data suggest, there is now robust experimental evidence showing that RAS is involved in muscle wasting and sarcopenia. Clinical studies in humans are scarce to date, but are emerging recently. Earlier studies performed in heart failure patients showed that RAS inhibition is associated with a lower prevalence of muscle wasting and higher muscle strength [73–75]. In older adults, several studies investigating the effects of RAS inhibition on muscle mass have inconsistent results. In a 3 year longitudinal study, continuous RAS inhibition resulted in a lower 3 year decline in knee extension strength and gait speed, compared to continuous/intermittent users of other antihypertensive drugs such as β-blockers, calcium channel blockers, thiazides, or α-blockers and those without any medication [76]. A cross-sectional study of community-dwelling, well-functioning older adults aged 70–79 years (*n*: 2431) has shown that ACE inhibitor use was associated with a larger lower extremity muscle mass compared to the use of thiazides, β-blockers and calcium channel blockers [77]. In a double-blind randomized controlled trial in elderly (mean age 78.7 years) perindopril treatment for 20 weeks improved 6 min walking distance vs. placebo [78]. Abadir et al. found that AT1R autoantibodies (reflecting a more active canonical ACE axis) was negatively associated with handgrip strength and gait speed in community-dwelling adults [79]. In middle-aged to elderly community residents (*n* = 344), 24 h urine excretion of angiotensinogen (as a measure of RAS activity) was independently and negatively associated with the thigh muscle cross-sectional area [80]. In a total of 272 community-dwelling adults with hypertension, sarcopenia was less prevalent in patients using ACE inhibitors vs. angiotensin II receptor blocker (ARB) and other antihypertensive drugs [81]. In a multi-center national study including patients with hypertension (*n* = 2613), ARB use was associated with higher muscle mass and gait speed values [82]. Although above studies are promising, there is no straightforward association between RAS blockage and muscle wasting. For example, Spira et al. showed that ACE-inhibition was not related with muscle quality or function [83]. Similarly, Cesari et al. did not report any improvement in muscle strength after 6 months of fosinopril use in elderly [84]. The use of ACE inhibitors was not associated with grip strength decline in healthy older people either [85]. These differences are possibly due to methodologic variations, patient heterogeneity, definition of sarcopenia and different assessment tools (e.g., appendicular skeletal muscle mass vs. anterior thigh muscle assessment), duration of interventions, and/or combinations of antihypertensive drugs [86].

Exercise is known as a preventive measure in reducing muscle atrophy [87, 88]. Exercise downregulates ACE/AngII/AT1R axis and upregulates ACE2/Ang 1–7/Mas axis [89–91]. It is shown that 10 weeks of aerobic exercise modulates ACE and β2-adrenergic receptor gene expression, decreasing AngII plasma levels [92]. In addition, AngII-induced effects on Akt pathway is altered by exercise via increased glucose uptake and insulin sensitivity [93]. The interactions between exercise, RAS inhibition and sarcopenia is not fully elucidated. One study showed that 4 mg perindopril for 20 weeks did not potentiate physical function improvement over regular exercise [94]. On the other hand, Buford et al. showed that in elderly individuals (aged 70–89 years, n = 424), 12 month of physical activity improved walking speed during a 400 m test in users of ACE inhibitors [95]. In young male army recruits, presence of ACE gene polymorphism insertion (I allele) -which is associated with lower ACE activity- is associated with a greater anabolic response and non-fat mass after 10 weeks of intensive physical training [96].

Type 2 diabetes mellitus (DM) is another common condition related with sarcopenia [97]. Reciprocally, aging, obesity, sarcopenia and chronic RAS activation, predate the development of T2DM [98, 99]. Yano et al. demonstrated that plasma renin activity was independently associated with appendicular skeletal muscle mass index in patients with DM [100]. Thus, the suppression canonical RAS operating through AT1R may be even more important in patients with DM since over activation of RAS activation is common in DM [101].

## Strategies to reduce muscle wasting

**Fig. 4:**
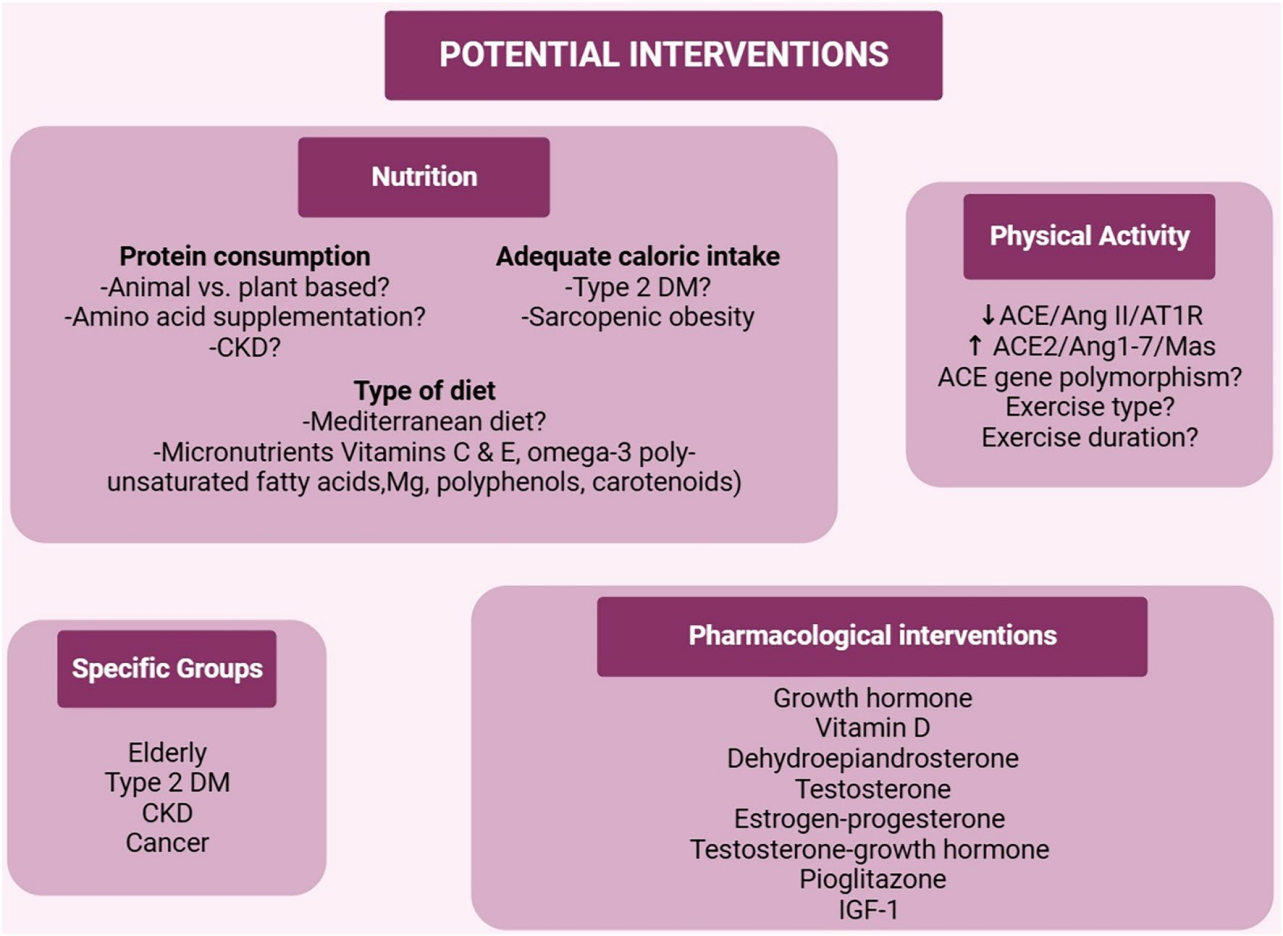
There are various potential ways to avoid muscle wasting, such as regular physical exercise, pharmacological treatment, nutritional interventions. The recommendations of increased protein intake and caloric intake may not be straightforward for patients with chronic kidney disease and diabetes, respectively. Also, the impact of Mediterranean diet on sarcopenia is not studied adequately. Physical activity is important to suppress canonical angiotensin converting enzyme (ACE)–Angiotensin Type 1 receptor (AT1R) axis and augment ACE2-Ang1-7-Mas axis, and thus is beneficial. However, it is not clear which type and duration of exercise is most efficient for the prevention of sarcopenia. Pharmacological interventions are important, but their interactions with renin-angiotensin system (RAS) should be studied further

While various potential ways to avoid muscle wasting are understood, such as regular physical exercise, pharmacological treatment, and nutritional interventions, there are still various knowledge gaps (Fig. 4). One of the most important ways to reduce muscle wasting is physical exercise. A recent meta-analysis (12 studies, 1347 older adults with sarcopenia) showed that resistance training improved handgrip strength and physical performance [102]. Another meta-analysis (including 42 randomized controlled trials including 3728 elderly participants) showed that in addition to nutrition, resistance and balance exercises were the most effective strategies for improving handgrip strength. [103]. The impact of exercise may be more important in the context of RAS inhibition, since a systemic review (11 randomized controlled trials including 375 individuals) demonstrated that plasma renin activity was reduced after exercise training [89]. In healthy men, aerobic physical exercise suppressed plasma renin and Ang II levels, and increased physical working capacity [104]. However, it is still not clear which type and duration of exercise induces RAS inhibition and improves muscle strength and function and needs further assessment.

Nutritional intervention is another option for sarcopenia management. In general, adequate protein consumption is mandatory for individuals with sarcopenia as protein is vital for muscle building and maintenance. In older individuals (70–79 years, *n* = 2066), the highest quintile of protein consumption (1.20 g/kg/day) results in 40% less loss of appendicular lean mass and total lean mass over a three-year period [105]. Furthermore, a recent meta-analysis showed that whey protein supplementation improves sarcopenia [106]. The most important question during protein supplementation is that whether animal or plant protein supplementation is more efficient in preventing sarcopenia. Recent findings suggest that animal proteins are more digestible than plant proteins, thus resulting in a greater amino acid availability with more stimulation of muscle protein synthesis. However, isolated plant proteins or modified plant proteins combined with amino acids can elicit comparable outcomes [107].

Adequate caloric intake is also important in the context of sarcopenia. In Korean National Health and Nutrition Examination Surveys between 2008 and2011 (*n* = 8165 participants) showed that sarcopenia is lower in individuals who consume more total energy and energy-producing nutrients [108]. Among elderly people, a meta-analysis showed that caloric intake is significantly lower in those with sarcopenia than without sarcopenia [109]. Indeed, in elderly people, an energy intake of approximately 30 kcal/kg body weight/day is recommended [110].

The type of diet may be also important. For example, Mediterranean diet has been positively associated with muscle mass and function [111], due to micronutrients (vitamins E, C, omega-3 poly-unsaturated fatty acids, magnesium, polyphenols and carotenoids) with their potential anti-inflammatory and anti-oxidant properties [112].

The specific relation between RAS and nutritional interventions are complex. Although adequate protein intake is suggested to ameliorate sarcopenia, this may not be proper in some certain conditions, such as in CKD. Previously, high protein intake has been shown to increase renal RAS [113], which is detrimental for sarcopenia. Importantly, low protein diets slow the progression of CKD, comprising more difficulty in managing sarcopenia in CKD patients [114]. Patients with type 2 DM are also a group of individuals in whom sarcopenia management is very important but is also difficult. Caloric restriction in obese individuals with DM are recommended [115]. However, body mass index (BMI) is not an ideal parameter for anthropometric evaluation. Yet, patients with type 2 DM may have sarcopenia despite increased BMI, a condition known as “sarcopenic obesity” [116]. Several studies have shown that in patients with type 2 DM, low energy intake is associated with sarcopenia [117, 118]. Thus, adequate caloric intake is important to prevent sarcopenia in type 2 DM patients, but close follow-up for obesity and blood sugars are mandatory. Given the fact that the peripheral RAS system suppression increases thermogenic capacity and caloric burn, the issues even get more complex [119]. Thus, studies are needed to highlight the interaction between RAS, caloric intake and sarcopenia.

Pharmacological intervention is another option for sarcopenia management, although no specific cure exists. Pharmacological alternatives include growth hormone, vitamin D, dehydroepiandrosterone, estrogen-progesterone combinations, testosterone-growth hormone combinations, pioglitazone, testosterone, IGF-1 [120]. With regard to our topic, vitamin D is especially important since vitamin D is a negative regulator of RAS. Vitamin D might reduce the activity of the renin gene promoter [121], and inhibit renin secretion and RAS activation throughout the body [122]. Vitamin D deficiency, on the other hand, is closely related with sarcopenia. In elderly, serum levels of vitamin D are independently associated with the loss of muscle mass and muscle strength [123–125]. However, the effects of oral vitamin D supplementation for the prevention of sarcopenia and frailty are equivocal, with some studies showing positive results [126–129], while others showing no change [130–133]. These findings confirm that more studies are needed to explore the vitamin D, RAS and sarcopenia axis.

## Areas of uncertainty and recommendations for future research

It is clear from the above discussion that RAS system potentially plays a role in muscle wasting. However, there are some gray zones that need to be addressed. One of the conflicting issues about RAS and muscle wasting is that whether the actions of AngII is direct or indirect is unknown. Some authors suggest that AngII could trigger muscle protein loss indirectly, since there is little expression of AngII receptors in muscle [31]. Conversely, others showed local RAS system is present in the muscle [134–138] and AngII have direct effects on myotubes [21]. Yoshida et al. demonstrated that AT1R was highly expressed in muscle satellite cells [13].

Another issue is the relative effects of AT1R vs. AT2R-Mas receptor on muscle wasting, which is not studied specifically. Whether the protection against muscle atrophy is due to a decrease of AT1R or to an increase of AT2R (which has opposing actions to AT1R) is of concern. AT1R blockade may increase AngII levels as a compensatory action. When ATR1 is blocked, the excess AngII may act through AT2R, which acts opposite to AT1R. Moreover, in the presence of ACE inhibitors, AngII is preferentially degraded by ACE2 through activation of Ang (1–7)/Mas receptor axis [139]. Indeed, as suggested above, Ang (1–7)/Mas axis has favorable actions in sarcopenia [2, 23, 24, 37]. However, these studies are limited and more studies are needed in this issue.

MicroRNAs (miRs) are also implicated in muscle wasting. In muscle cells, miR-1, miR-133a, miR-133b, and miR-206 direct myoblasts to differentiate into mature myotubes, which are organized into myofibers [140]. Wang et al. demonstrated that in chronic kidney disease (CKD), miR-29a and miR-29b down-regulation suppressed myogenesis [141]. Xu et al. demonstrated that miR-486 prevented muscle wasting in mice [142]. As microRNAs play a role in modulation of RAS [143, 144], studies will be of importance whether modulation of RAS by miRs has impacts on muscle wasting.

Muscle fiber atrophy may be specific in different conditions and depends on fiber type. For example, type I fibers atrophy more during inactivity and denervation, whereas type II fibers atrophy more in cases of cancer cachexia, diabetes, heart failure and ageing [145]. Zhang et al. showed that AngII infusion in mice shifted the fiber distribution toward type IIB fibers, but there were no differences in type I or IIA fibers, implying that type IIB fibers may be more susceptible to AngII-induced proteolysis [31]. Kadaguchi et al. demonstrated that 1-week AngII treatment in mice resulted in decreased of Type I fibers and increased Type IIb fibers [43]. The clinical relevance of these findings is not studied in detail and future research is needed in this issue.

It is critical to understand the impact of RAS inhibition on specific patient groups. Elderly people and people with diabetes, CKD, cancer have high prevalence of sarcopenia [146–148]. Most of the studies regarding RAS inhibition and sarcopenia involved elderly population. Although sarcopenia is highly prevalent in cancer patients, to the best of our knowledge, there is no specific study addressing the impact of RAS inhibition on sarcopenia in patients with cancer. The studies in CKD are also scarce. A very recent study involving patients with end-stage kidney disease showed that ACE inhibitor use prevented the decrease of skeletal muscle mass (calculated by modified creatinine index) induced by β-blockers [149].

To note, not all the studies have demonstrated beneficial effects of RAS blockage on muscle strength [150], gait performance [151] and muscle quality [83]. The exact mechanisms of these discrepancies are not known, but could be explained by the difference expression of Ang II receptors in myoblasts, myotubes and mature myofibers [136] and difference in muscle microenvironment [64]. Notably, AT1R signaling reactions are tissue specific. Indeed, knockout of AT1R in different tissues reveals that second messenger signaling pathways of AT1R differ among cell types and therefore, investigating AT1R signaling in a tissue specific manner may be of importance [152].

The relationship between mineralocorticoid receptor antagonists (MRAs) is also worth noting although the studies are rare. In alcoholic liver cirrhosis, spironolactone treatment increased muscle mass, strength and muscle magnesium levels [153]. It has also been suggested that aldosterone suppressed insulin-mediated glucose uptake in skeletal muscle and increase oxidative stress. In insulin resistance rats, Lastra et al. showed that mineralocorticoid activation increased oxidase stress, and reduction in expression of IRS-1 and GLUT 4 levels in the soleus muscle. However, low-dose spironolactone improved muscle insulin signaling and reduced oxidative stress with no changes in blood pressure [154]. In patients with heart failure, use of MRAs (spironolactone and eplerenone) was associated with lower appendicular skeletal muscle mass index and higher plasma renin activity in patients not receiving ACEi/ARB but not in patients receiving ACEi/ARB suggesting that MRA use without concurrent RAS inhibition, may contributing to upregulation of angiotensin II signaling, may be associated with reduction in muscle mass [155]. Thus more studies are needed to highlight the relationship between MRA use and muscle wasting. Of note, we did not find any study regarding a use of new mineralocorticoid receptor antagonist finerenone with muscle wasting/sarcopenia. Lastly, more studies are needed to investigate the relationship between exercise, muscle wasting and RAS.

## Conclusions

RAS system plays a role in muscle atrophy by a variety of mechanisms. While RAS actions through ACE/AngII/AT1R axis is considered as detrimental, the ACE2/Ang 1–7/Mas axis is considered as beneficial. However, the preclinical and clinical data are limited and there are various knowledge gaps. Human clinical studies regarding RAS and sarcopenia are scarce but are recently emerging. Especially, prevalence of sarcopenia is high in specific conditions such as in elderly, in patients with cancer, type 2 DM, heart failure and CKD. Specific recommendations for management of sarcopenia in these patient groups are mostly lacking. Evidence-based data are scarce and management is mostly guided by expert opinions. This may be not only due to lack of controlled studies, but also due to complexity and conflicting issues such as caloric intake management in type 2 DM and protein supplementation in CKD, and their relationship with RAS. The data about the pharmacologic interventions in these specific conditions and their impacts on RAS are also lacking, which need to be highlighted in future studies.

## Data Availability

This article does not involve any generation or analysis of datasets, therefore, data sharing is not relevant**.**

## Abbreviations

ACE: Angiotensin-converting enzyme
AMPK: AMP-activated protein kinase
AngII: Angiotensin II
AT1R: Angiotensin type 1 receptor
AT2R: Angiotensin type 2 receptor
CTGF: Connective tissue growth factor
ERK: Extracellular-signal-regulated kinase
FOX: Forkhead box protein
GSK3β: Glycogen synthase kinase-3 beta
IGF-1: Insulin-like growth factor 1
IRS-1: Insulin receptor-substrate-1
MAPK: Mitogen-activated protein kinase
mTOR: Mammalian target of rapamycin
MuRF1: Muscle RING-finger protein-1
MyoD: Myoblast determination protein-1
NF-κB: Nuclear factor kappa B
PI3K: Phosphatidylinositol 3-kinases
PKD1: Protein kinase D1
RAS: Renin angiotensin system
ROS: Reactive oxygen species
SOCS: Suppressor of cytokine signalling
STAT: Signal transducer and activator of transcription
TFEB: Transcription factor EB
TGF-β: Transforming growth factor beta
UPS: Ubiquitin–proteasome system
NPY: Neuropeptide Y
